# Near- and medium-term hourly morphed mean and extreme future temperature datasets for Jyväskylä, Finland, for building thermal energy demand simulations

**DOI:** 10.1016/j.dib.2021.107209

**Published:** 2021-06-10

**Authors:** Jari Pulkkinen, Jean-Nicolas Louis

**Affiliations:** University of Oulu, Faculty of Technology, Water, Energy and Environmental Engineering, PO Box 4300, Oulu FI-90014, Finland

**Keywords:** Climate change, Future outdoor temperature, Morphing, Building Energy Simulation

## Abstract

Near- and medium-term hourly morphed outdoor temperature files were created for Jyväskylä, Finland, to be used in building energy simulation. These future outdoor temperature files were created according to a statistical down-scaling method, morphing, which utilizes both hourly baseline data, and monthly and daily future climate projections. The used baseline data included hourly test reference year and typical meteorological year data to represent a “typical” climate year, and were appended with weather files created based on the coldest and warmest near Januaries to represent extreme weather files. Climate change data included climate change projection data from 2 different data repositories for either results from Global Climate Model or Regional Climate Model simulations for RCP2.6, RCP4.5 and RCP8.5 climate change scenarios. Morphing all 5 different baseline scenarios with each of the available climate scenarios creates 25 future outdoor temperature files for 2030 (near-term) and 25 files for 2050 (medium-term). The created files were used in [1] to simulate future thermal energy demand in buildings.

**Specifications Table**

**Table:** 

Subject	Climatology
Specific subject area	Climatology; Statistical down-scaling; Morphing; Future outdoor temperature; Building Energy Simulation
Type of data	Table
	Graph
	CSV -files
How data were acquired	Collection of baseline data from public repositories from Finnish Meteorological Institute (FMI) and Climate.OneBuilding.org;
	Climate change data from public repositories from The European Climatic Energy Mixes (ECEM) Demonstrator by Copernicus Climate Change Service (C3S) and FMI’s down-scaled CMIP5 data from https://www.csc.fi/paituli;
	Future weather files created with morphing method described in the article
Data format	Analyzed
	Processed
Parameters for data collection	The data was collected according to the location (city, cluster and country), suitable time-periods (historical, future for 2030 and 2050), climate change scenario (RCP2.6, RCP4.5, RCP8.5), different climate change repository for data created with different climate models (GCMs, RCMs), and baseline scenarios for typical year (mean), and warm and cold Januaries (extreme) for different repositories
Description of data collection	Data was collected according to the given parameters from publicly available data repositories, and then statistically down-scaled to create future outdoor temperature files
Data source location	Primary data sources:
	Baseline:
	Climate.OneBuilding.org data repository:
	Location: Jyväskylä, Central Finland, Finland;
	Datafiles: FIN_CF_Jyvaskyla.AP.029350_TMYx.2004-2018.epw &
	FIN_CF_Jyvaskyla.AP.029350_TMYx.epw
	Finnish Meteorological Institute’s Test Reference Year 2012:
	Location: Area III
	Datafile from: https://www.ilmatieteenlaitos.fi/energialaskennan-testivuodet-nyky (In Finnish);
	Finnish Meteorological Institute’s Open data repository:
	https://en.ilmatieteenlaitos.fi/open-data;
	Instantaneous Observations: Air Temperature, 1 hour;
	Time Period: 1.1.2008-31.12.2008 & 1.1.2010-31.12.2010;
	Location: Jyväskylä Lentoasema (Jyväskylä Airport);
	Climate change:
	Gridded ensemble CMIP5 for Finland:
	Monthly mean precipitation and temperature predictions, 1975-2085, 10 km:
	http://urn.fi/urn:nbn:fi:csc-kata20171023170231365082
	Daily mean temperature predictions, 1981-2100:
	http://urn.fi/urn:nbn:fi:csc-kata20180918114340806244
	Daily minimum temperature predictions, 1981-2100:
	http://urn.fi/urn:nbn:fi:csc-kata20180918114342932242
	Daily maximum temperature predictions, 1981-2100:
	http://urn.fi/urn:nbn:fi:csc-kata20180918103914033689
	The European Climatic Energy Mixes (ECEM) demostrator data for South Finland cluster:
	http://ecem.wemcouncil.org;
	Time Period: Projections;
	Variables: Air Temperature;
	Temporal Resolution: Daily & Monthly;
	Statistics: Absolute Values;
	Climate Model: RCM1-7 (daily) & Ensemble Mean (monthly)
Data accessibility	Data is available at the following data repository:
	Repository name: Zenodo.org
	Dataset Name: Long-term heat demand scenarios under climate change utilising a stochastic dynamic building stock model: Morphed hourly outdoor temperatures for Jyvaskyla for 2030 and 2050
	Data identification number: https://doi.org/10.5281/zenodo.4275759
	Direct URL to data: https://doi.org/10.5281/zenodo.4275759
Related research article	Hietaharju, P., Louis, J.-N., Pulkkinen, J. & Ruusunen, M. A stochastic dynamic building stock model for determining long-term district heating demand under future climate change Applied Energy. 295 (2021) 116962. https://doi.org/10.1016/j.apenergy.2021.116962

## Value of the Data

• Assessment of future thermal energy demand for buildings requires hourly outdoor temperature data to be used on building energy simulations.
• This data can benefit anyone interested in simulating future thermal demand of buildings on Jyväskylä, Finland or Nordic area.
• This data can be directly used as an input data to building energy simulations, which require future hourly outdoor temperature files.
• This data can help in assessing future energy demands in local, regional or country level on typical and extreme weathers through utilization in building energy simulation.
• Additionally, the data can be used to assess any future goals that require energy demand or other outputs from building energy simulations for mean and extreme weather years.
• Inclusion of extreme weather years allows studying the system level design for energy systems dependable on them, e.g. on district heating network design.

## 1 Data Description

### 1.1 Data for current climate

There are 3 datasets containing averaged outdoor temperature (*T*) data to represent averaged weather conditions in different time periods for the Jyväskylä, Finland area. These datasets are presented in Table 1 showing the averaged time period and the source of original data. Additionally, to introduce the extreme heating season weather conditions, a representative cold and warm January weather data are included and presented in Table 1.

**Table 1:** The outdoor temperature data available for use in building heating energy simulation.

Sce.	Name of Weather file	Averaged Time Period	Place	Data type	Source of data
S2	Typical Meteorological Year (TMY)	1952–2019	Jyväskylä AP	EPW-file	[2]
S3	Typical Meteorological Year 2004–2018 (TMY2004-2018)	2004–2018	Jyväskylä AP	EPW-file	[2]
S4	Test Reference Year 2012 (TRY2012)	1980–2009	Area III	prn xls -files	[3]
S1	Cold2010	2010	Jyväskylä AP	xls csv -files	[4]
S5	Warm2008	2008	Jyväskylä AP	xls csv -files	[4]

All the data represented in Table 1 is presented in hourly time-scale and expands to a full year in length. The Typical Meteorological Year (TMYx) data is created according to TMY/ISO 15927-4:2005 methodology from hourly observations of the mentioned observation station to present averaged weather conditions for the time period from Table 1 by Lawrie and Crawley [2]. Test Reference Year 2012 (TRY2012) is created according to the same principle and standard as TMYx considering only different averaging period and a larger geographical area than a single observation station. TRY2012 are created by dividing Finland into 4 meteorological areas, which differ from each other, but are considered to have similar enough climate inside them. Jyväskylä belongs to Area III which consists mainly areas located in central Finland [3]. To improve the validity of future simulations, representative cold (Cold2010) and warm (Warm2008) Januaries were added to the dataset to present extreme weather conditions. The selection of these weather data was based on the lowest and highest average temperatures in January in Jyväskylä Airport (Jyväskylä AP) observation station from Finnish Meteorological Institute [4].

The characteristics of these weather datasets are presented in Table 2 including monthly average temperatures, mean differences between daily maximum and minimum temperatures on every month and the standard deviation of daily mean temperatures in each month.

**Table 2:** Historical mean monthly temperature, mean change between daily maximum and minimum temperatures, and monthly standard deviation of daily mean temperatures in the selected datasets [2], [3], [4].

Month	Average Temperature [°C]	Max Min Change [°C]	Std, daily mean temp [-]	Average Temperature [°C]	Max Min Change [°C]	Std, daily mean temp [-]
	TMY			TMY2004–2018		
January	−8.1	6.3	6.6	−7.3	6.3	5.5
February	−7.6	6.5	5.4	−7.1	6.7	5.7
March	−3.7	9.7	3.4	−3.7	9.7	3.4
April	1.9	7.8	3.4	2.9	10.0	3.1
May	9.3	9.9	3.8	9.5	11.2	3.5
June	13.4	9.3	2.9	13.6	9.5	2.4
July	16.5	9.0	2.1	15.7	9.7	2.8
August	14.3	9.0	2.8	14.3	8.5	2.2
September	8.9	9.3	4.4	10.3	9.2	2.7
October	4.2	4.7	4.6	4.3	6.8	4.1
November	−0.9	3.3	5.2	0.6	3.4	2.8
December	−4.1	7.0	6.0	−3.1	6.1	4.0

	TRY2012			Cold2010		
January	−8.0	5.7	6.6	−15.8	7.7	6.1
February	−7.1	6.2	5.6	−12.0	8.2	6.5
March	−3.5	5.7	4.3	−4.7	9.7	5.4
April	2.4	9.1	3.5	3.4	8.5	1.7
May	8.8	11.4	4.3	10.9	10.5	5.6
June	13.4	8.9	4.3	13.3	11.5	2.6
July	15.8	8.9	2.8	21.1	11.6	2.9
August	13.8	8.4	3.7	15.6	10.2	4.5
September	9.2	7.3	3.0	9.4	8.2	2.8
October	4.1	5.2	2.5	3.2	5.1	3.3
November	−1.7	3.7	3.2	−4.5	3.3	6.3
December	−5.9	5.8	6.5	−14.0	6.2	6.0

	Warm2008			-		
January	−3.1	4.0	4.1	-	-	-
February	−2.7	5.7	3.2	-	-	-
March	−3.5	6.5	4.3	-	-	-
April	3.6	9.9	3.3	-	-	-
May	8.5	12.2	4.1	-	-	-
June	12.8	11.2	2.8	-	-	-
July	15.0	9.9	2.4	-	-	-
August	12.5	7.6	2.4	-	-	-
September	7.3	8.9	2.5	-	-	-
October	5.6	5.5	1.9	-	-	-
November	0.2	4.6	3.2	-	-	-
December	−1.4	3.1	2.3	-	-	-

The data from Table 2 shows similarities in average outdoor temperatures on TMYx and TRY2012 weather files, but some differences on the temperature variation. The Cold2010 weather file clearly has the lowest average temperature during the heating season, while the Warm2008 file has the highest. The average heating season (October-April) temperatures of each of the weather file are presented in Table 3.

**Table 3:** Average temperature of each dataset in heating periods [2], [3], [4].

	TMY	TMY2004-2018	TRY2012	Cold2010	Warm2008
Jan-Apr [°C]	−4.34	−3.77	−4.04	−7.25	−1.42
Oct-Dec [°C]	−0.28	0.61	−1.19	−5.11	1.47
Whole Heating Season [°C]	−2.58	−1.87	−2.80	−6.32	−0.16

### 1.2 Data for climate change

Applying data from climate change projections, 2 main aspects to acknowledge are the used Climate models, which are either Global Climate Models (GCMs) or Regional Climate Models (RCMs), and the climate change scenarios describing the future Greenhouse gas (GHG) emissions levels, both which combined will give the projected future weather variables. The current climate change data projections are conducted under Representative Concentration Pathway (RCP) scenarios, presenting different GHG emission pathways for the future based on their ending radiate forcing value for 2100. The scenarios currently include RCP2.6, RCP4.5, RCP6.0 and RCP8.5 scenarios, presented from lowest to highest radiate forcing value for 2100 [5]. The second main aspect was the used climate model, which depend on the availability and accuracy of data as well as the climate scenario. For Jyväskylä, Finland, 2 data sources were selected:

- Results from Global Climate Models (GCMs) conducted originally under Coupled Model Intercomparison Project 5 (CMIP5) [6], from which results for Finland are gathered and presented in [7]. Modified results for 10 x 10 km spacial resolution based on a baseline from [8] are available for RCP2.6, RCP4.5 and RCP8.5 scenarios on monthly mean outdoor temperature values in [9], daily mean outdoor temperature values in [10], daily maximum outdoor temperature value in [11] and daily minimum outdoor temperature value in [12].
- Regional Climate Models (RCMs) are dynamically down-scaled data from GCMs, presenting simulation results on finer spacial and time resolutions [13]. European Climatic Energy Mixes (ECEM) demonstrator from Copernicus Climate Change Service (C3S)^1^ provide ensemble and individual results from RCMs on country and cluster level. The data for outdoor temperature projections are available on daily, monthly, seasonal and yearly level on RCP4.5 and RCP8.5 scenarios.

A summary of the available climate change projection data is presented on Table 4. In Table 4 $T_{daily,max}$ and $T_{daily,min}$ represent the availability of daily maximum and minimum temperature data, respectively, $T_{daily,mean}$ represents availability of daily mean temperature data, and $T_{monthly,mean}$ represents the availability of monthly average temperature data.

**Table 4:** Description of the available data from the climate models.

	RCP2.6	RCP4.5	RCP8.5	$T_{daily,max}$	$T_{daily,min}$	$T_{daily,mean}$	$T_{monthly,mean}$
GCMs	x	x	x	x	x	x	x
RCMs	-	x	x	-	-	x	x

The climate change projection data from both GCMs and RCMs for Jyväskylä is presented in Table 5 for 2030 and in Table 6 for 2050. The GCM results are ensemble on country level, and RCM results are ensemble for South-Finland cluster. The average monthly temperature $T_{mean}$ is a 30-year average value from monthly mean datasets, whereas the mean daily max min change $\Delta T_{max,min}$ and daily mean temperature standard deviations $\sigma_{T}$ are as well averaged over 30-year period, but use daily datasets.

**Table 5:** Projected mean monthly temperatures $T_{mean}$, changes between daily max and min temperatures $\Delta T_{max,min}$ and standard deviation of monthly daily mean temperatures $\sigma_{T}$ in RCP2.6, RCP4.5 and RCP8.5 climate scenarios from RCM (ECEM) and GCM results [9], [10], [11], [12] in 2030.

RCP2.6						
	RCM			GCM		
Month	$T_{mean}$ [°C]	$\Delta T_{max,min}$ [°C]	$\sigma_{T}$ [-]	$T_{mean}$ [°C]	$\Delta T_{max,min}$ [°C]	$\sigma_{T}$ [-]

January	-	-	-	−6.0	5.6	5.9
February	-	-	-	−6.6	6.5	5.1
March	-	-	-	−2.1	7.3	3.7
April	-	-	-	3.7	8.9	3.5
May	-	-	-	10.4	10.8	3.5
June	-	-	-	15.0	9.8	4.4
July	-	-	-	17.9	9.8	1.7
August	-	-	-	15.6	8.6	1.1
September	-	-	-	10.4	7.2	2.5
October	-	-	-	5.1	5.0	3.7
November	-	-	-	−0.1	4.0	3.2
December	-	-	-	−4.3	5.1	5.9

RCP4.5						

	RCM			GCM		
January	−6.1	-	5.2	−5.8	5.5	5.9
February	−5.7	-	4.8	−6.5	6.6	5.1
March	−1.3	-	3.4	−1.9	7.3	3.7
April	4.3	-	2.8	4.0	8.9	3.4
May	10.4	-	3.4	10.6	11.0	3.6
June	15.2	-	2.9	15.1	9.9	4.3
July	17.6	-	2.1	17.9	9.6	1.7
August	15.8	-	2.4	15.7	8.6	1.1
September	10.5	-	2.8	10.5	7.2	2.6
October	5.3	-	3.4	5.1	5.0	3.7
November	−0.2	-	3.5	0.1	3.9	3.1
December	−4.6	-	4.3	−4.1	5.0	5.7

RCP8.5						

	RCM			GCM		
January	−6.4	-	5.3	−5.8	5.3	5.9
February	−5.3	-	4.6	−6.1	6.3	5.0
March	−0.6	-	3.1	−1.9	7.1	3.6
April	4.5	-	2.9	4.0	8.9	3.5
May	10.5	-	3.2	10.6	10.9	3.5
June	15.4	-	2.9	15.2	9.8	4.4
July	17.7	-	2.2	18.2	9.6	1.8
August	15.8	-	2.5	15.8	8.6	1.2
September	10.7	-	2.7	10.7	7.1	2.5
October	5.6	-	3.2	5.4	5.0	3.7
November	0.3	-	3.4	0.4	3.9	3.1
December	−4.2	-	4.3	−3.8	4.9	5.7

**Table 6:** Projected mean monthly temperatures $T_{mean}$, changes between daily max and min temperatures $\Delta T_{max,min}$ and standard deviation of monthly daily mean temperatures $\sigma_{T}$ in RCP2.6, RCP4.5 and RCP8.5 climate scenarios from RCM (ECEM) and GCM results [9], [10], [11], [12] in 2050.

RCP2.6						
	RCM			GCM		
Month	$T_{mean}$ [°C]	$\Delta T_{max,min}$ [°C]	$\sigma_{T}$ [-]	$T_{mean}$ [°C]	$\Delta T_{max,min}$ [°C]	$\sigma_{T}$ [-]
January	-	-	-	−5.5	5.3	6.2
February	-	-	-	−6.2	6.2	2.6
March	-	-	-	−1.6	7.3	4.2
April	-	-	-	4.2	8.9	4.1
May	-	-	-	10.6	10.8	2.6
June	-	-	-	15.2	9.8	3.3
July	-	-	-	18.2	9.7	1.6
August	-	-	-	15.9	8.6	2.8
September	-	-	-	10.7	7.2	2.5
October	-	-	-	5.5	5.0	2.8
November	-	-	-	0.2	3.9	4.8
December	-	-	-	−3.8	4.9	2.8

RCP4.5						

	RCM			GCM		
January	−5.6	-	5.0	−4.9	5.1	6.1
February	−4.7	-	4.4	−5.5	6.1	2.6
March	−0.3	-	3.0	−1.0	7.1	3.9
April	5.0	-	2.9	4.7	8.9	4.0
May	11.0	-	3.4	11.2	10.8	2.7
June	15.5	-	2.8	15.7	9.8	3.3
July	18.0	-	2.2	18.5	9.6	1.6
August	16.2	-	2.4	16.3	8.7	2.7
September	11.0	-	2.6	11.0	7.3	2.5
October	5.7	-	3.3	5.8	5.0	2.8
November	0.4	-	3.4	0.7	3.8	4.8
December	−3.7	-	4.1	−3.1	4.6	2.8

RCP8.5						

	RCM			GCM		
January	−5.3	-	5.0	−3.8	4.7	5.5
February	−4.2	-	4.2	−4.5	5.6	2.4
March	0.5	-	2.9	−0.5	6.8	3.9
April	5.6	-	2.8	5.2	8.9	3.9
May	11.2	-	3.2	11.4	10.7	2.7
June	16.0	-	2.8	16.1	9.7	3.3
July	18.6	-	2.2	19.2	9.6	1.6
August	16.6	-	2.4	16.9	8.6	2.7
September	11.6	-	2.6	11.8	7.3	2.5
October	6.7	-	3.2	6.5	4.9	2.7
November	1.0	-	3.5	1.6	3.7	4.5
December	−3.2	-	4.0	−2.2	4.5	2.5

### 1.3 Morphed outdoor temperatures

**Fig. 1:**
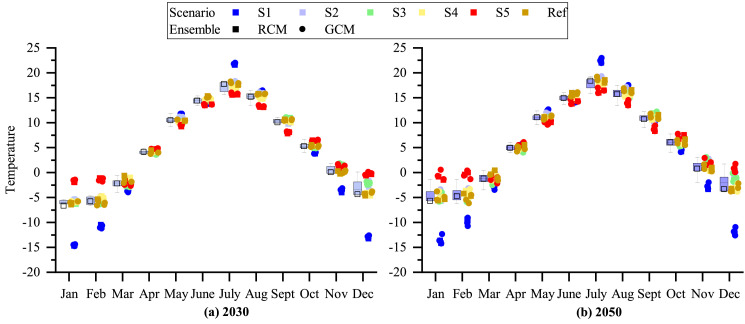
Distribution of the morphed temperature data and comparison with their monthly mean temperature against input data on climate change (Ref) for 2030 and 2050.

**Fig. 2:**
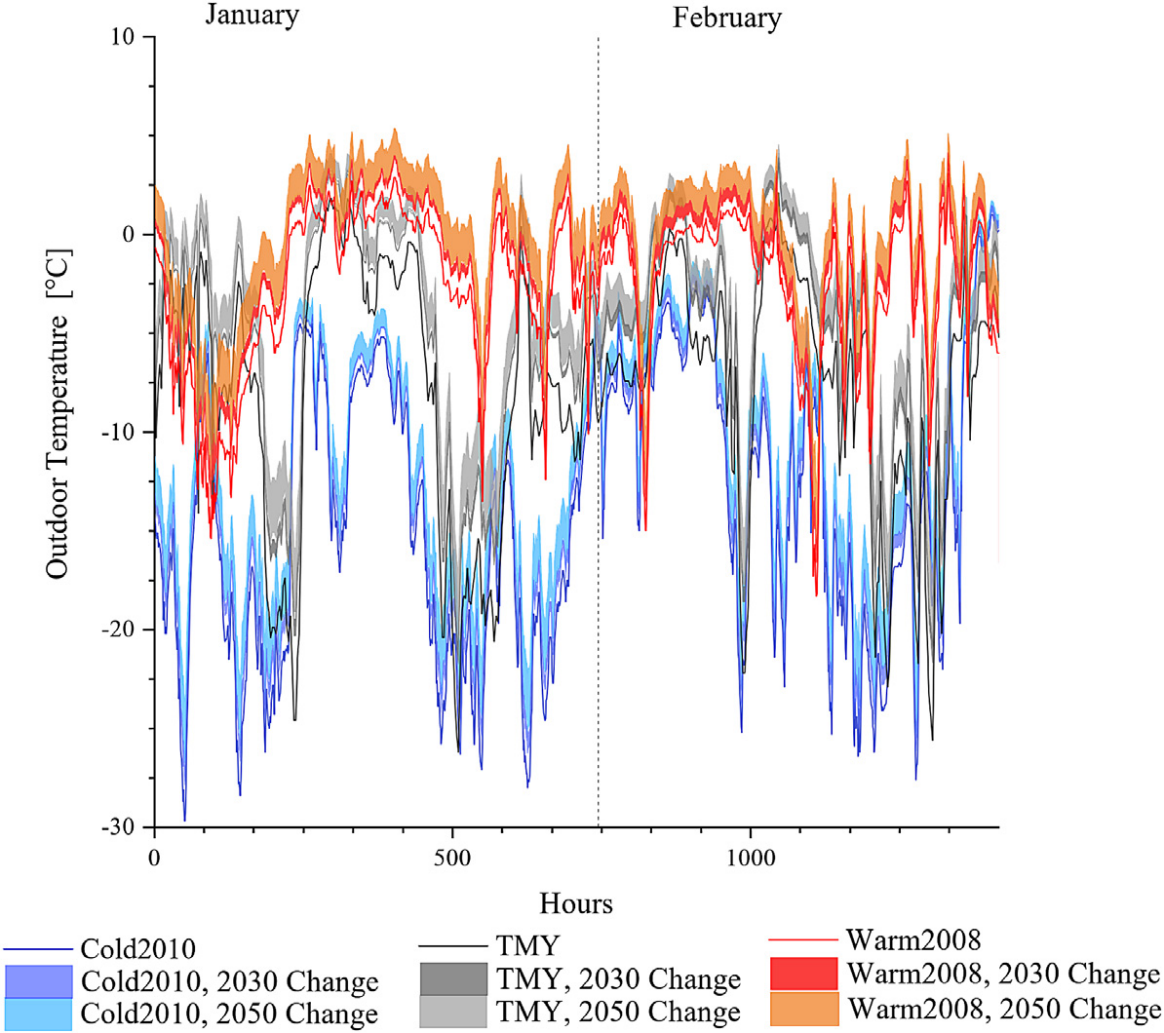
The changes in Cold2010, Warm2008 and TMY weather files by 2030 and 2050 in January and February.

**Fig. 3:**
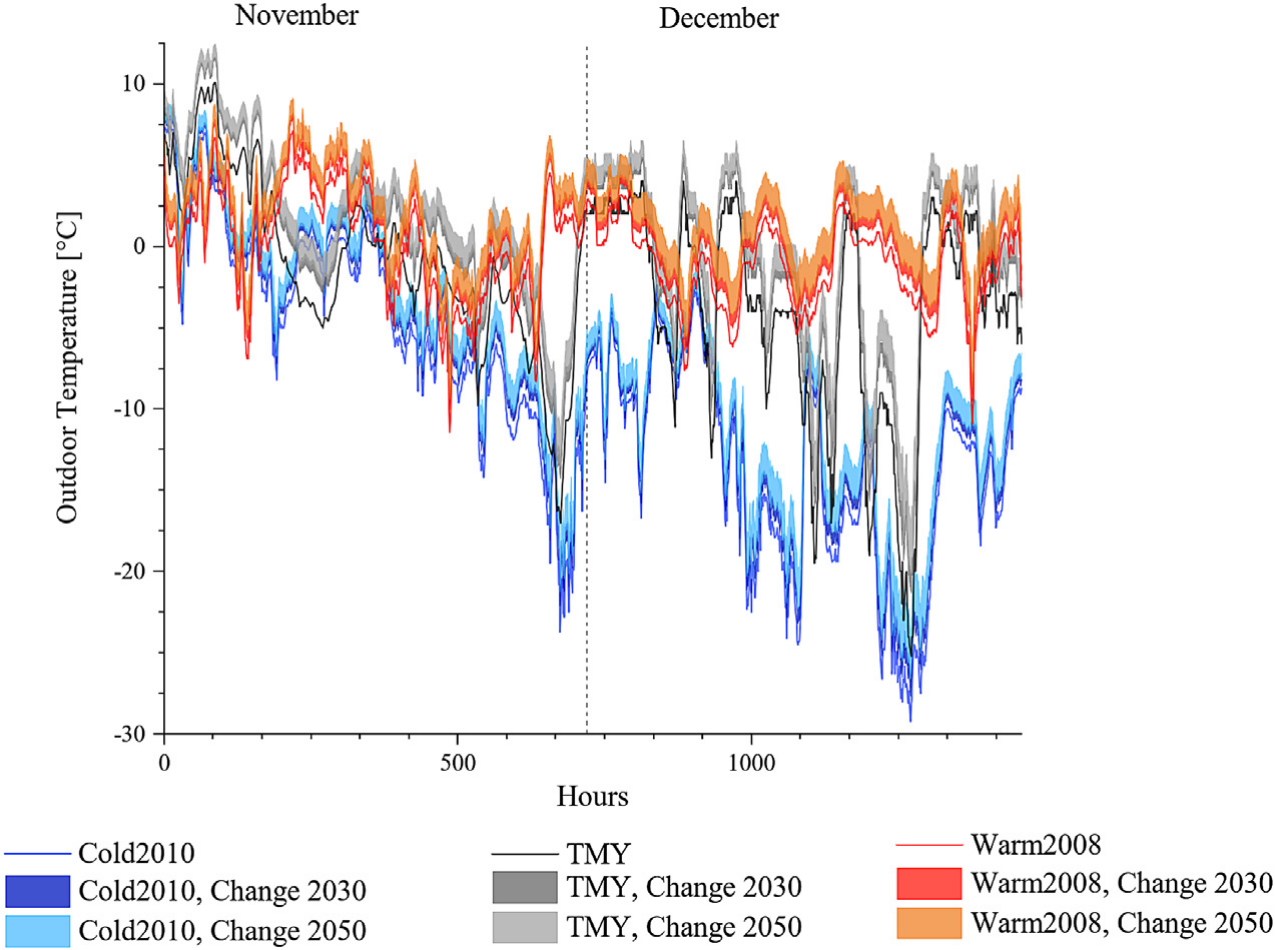
The changes in Cold2010, Warm2008 and TMY weather files by 200 and 2050 in November and December.

The future outdoor temperature is created with morphing method [14] from the baseline weather data and the climate change projections as described in Section 2.1. This results in 25 weather files for 2030 and 25 weather files for 2050. The morphed outdoor temperatures present a scenario of how the existing baseline scenario would look like in 2030 or 2050 by the changes happening according to the climate change scenario and climate model simulation results. Examples for the outdoor temperature change for January-February and November-December for Cold2010, TMY and Warm2008 scenarios are presented in Figs. 2 and 3 respectively. Furthermore, the average statistical results for the mean monthly temperatures and associated standard deviations are provide for each weather scenario (S1–S5, as listed in Table 1), RCPs and climatic projection RCM and GCM in Table 7, Table 8, Table 9, Table 10, Table 11. A comparison with the reference tables on climate change Tables 5 and 6 is presented in Fig. 1.

**Table 7:** Morphed mean monthly temperatures $T_{mean}$, and standard deviation of monthly daily mean temperatures $\sigma_{T}$ in RCP2.6 GCM (Paituli) in 2050. Complete hourly dataset available in [15].

RCP2.6 - GCM										
	S1		S2		S3		S4		S5	
	$T_{mean}$	$\sigma_{T}$	$T_{mean}$	$\sigma_{T}$	$T_{mean}$	$\sigma_{T}$	$T_{mean}$	$\sigma_{T}$	$T_{mean}$	$\sigma_{T}$
	[${}^{\circ }$C]	[${}^{\circ }$C]	[${}^{\circ }$C]	[${}^{\circ }$C]	[${}^{\circ }$C]	[${}^{\circ }$C]	[${}^{\circ }$C]	[${}^{\circ }$C]	[${}^{\circ }$C]	[${}^{\circ }$C]
Jan	−14.3	6.0	−5.1	5.9	−5.8	5.4	−5.5	5.9	−1.3	3.8
Feb	−10.8	6.5	−4.8	5.2	−5.9	5.6	−5.0	5.3	−1.3	3.5
Mar	−3.5	6.3	−1.3	4.6	−2.5	4.8	−1.6	4.5	−2.2	4.8
Apr	4.5	3.5	4.2	4.5	4.0	5.0	4.3	4.7	4.9	4.9
May	11.9	6.6	11.2	5.0	10.4	5.4	10.4	5.8	9.6	5.7
Jun	14.1	4.5	15.1	4.3	14.4	4.2	14.8	5.3	13.7	4.5
Jul	21.9	4.8	18.3	3.9	16.5	4.4	17.2	4.3	15.9	4.2
Aug	16.5	5.7	16.1	4.3	15.1	3.5	15.3	4.7	13.5	3.4
Sep	10.3	4.1	10.6	5.4	11.1	4.2	10.6	4.1	8.3	4.0
Oct	4.1	3.7	6.0	4.8	5.2	4.7	5.6	3.2	6.6	2.7
Nov	−3.3	6.0	1.4	4.9	1.8	2.9	0.2	3.2	1.6	3.2
Dec	−12.6	5.8	−1.6	5.8	−1.8	4.2	−3.9	6.0	0.1	2.4

**Table 8:** Morphed mean monthly temperatures $T_{mean}$, and standard deviation of monthly daily mean temperatures $\sigma_{T}$ in RCP4.5 GCM (Paituli) in 2050. Complete hourly dataset available in [15].

RCP4.5 - GCM										
	S1		S2		S3		S4		S5	
	$T_{mean}$	$\sigma_{T}$	$T_{mean}$	$\sigma_{T}$	$T_{mean}$	$\sigma_{T}$	$T_{mean}$	$\sigma_{T}$	$T_{mean}$	$\sigma_{T}$
	[${}^{\circ }$C]	[${}^{\circ }$C]	[${}^{\circ }$C]	[${}^{\circ }$C]	[${}^{\circ }$C]	[${}^{\circ }$C]	[${}^{\circ }$C]	[${}^{\circ }$C]	[${}^{\circ }$C]	[${}^{\circ }$C]
Jan	−13.6	5.8	−4.5	5.7	−5.2	5.2	−4.9	5.7	−0.7	3.7
Feb	−10.1	6.2	−4.2	5.1	−5.3	5.4	−4.4	5.2	−0.6	3.4
Mar	−2.8	6.1	−0.7	4.5	−1.8	4.6	−1.0	4.3	−1.5	4.6
Apr	5.1	3.4	4.7	4.5	4.6	5.0	4.8	4.7	5.5	4.9
May	12.4	6.5	11.7	4.9	10.9	5.3	10.9	5.8	10.1	5.7
Jun	14.5	4.5	15.5	4.3	14.9	4.1	15.3	5.3	14.2	4.5
Jul	22.4	4.8	18.7	3.9	17.0	4.4	17.6	4.2	16.4	4.2
Aug	16.8	5.7	16.5	4.3	15.5	3.5	15.7	4.8	13.8	3.5
Sep	10.6	4.1	11.0	5.5	11.4	4.3	11.0	4.1	8.6	4.1
Oct	4.5	3.7	6.4	4.7	5.6	4.6	6.0	3.2	7.1	2.6
Nov	−2.8	5.8	1.9	4.7	2.2	2.8	0.7	3.1	2.0	3.1
Dec	−11.9	5.5	−0.9	5.5	−1.1	4.1	−3.2	5.7	0.8	2.3

**Table 9:** Morphed mean monthly temperatures $T_{mean}$, and standard deviation of monthly daily mean temperatures $\sigma_{T}$ in RCP8.5 GCM (Paituli) in 2050. Complete hourly dataset available in [15].

RCP8.5 - GCM										
	S1		S2		S3		S4		S5	
	$T_{mean}$	$\sigma_{T}$	$T_{mean}$	$\sigma_{T}$	$T_{mean}$	$\sigma_{T}$	$T_{mean}$	$\sigma_{T}$	$T_{mean}$	$\sigma_{T}$
	[${}^{\circ }$C]	[${}^{\circ }$C]	[${}^{\circ }$C]	[${}^{\circ }$C]	[${}^{\circ }$C]	[${}^{\circ }$C]	[${}^{\circ }$C]	[${}^{\circ }$C]	[${}^{\circ }$C]	[${}^{\circ }$C]
Jan	−13.6	5.7	−3.4	5.2	−4.0	4.9	−3.8	5.2	0.5	3.5
Feb	−9.3	5.8	−3.2	4.7	−4.3	5.1	−3.3	4.8	0.4	3.1
Mar	−2.0	5.4	−0.2	4.3	−1.3	4.5	−0.5	4.1	−1.0	4.4
Apr	5.4	3.2	5.2	4.4	5.1	4.9	5.4	4.7	6.1	4.9
May	12.4	6.1	12.0	4.9	11.2	5.2	11.2	5.7	10.4	5.6
Jun	14.7	4.5	16.0	4.3	15.3	4.1	15.7	5.2	14.6	4.5
Jul	22.5	4.8	19.3	3.9	17.5	4.4	18.2	4.2	17.0	4.1
Aug	16.9	5.4	17.2	4.3	16.2	3.5	16.3	4.8	14.5	3.5
Sep	11.1	3.6	11.8	5.4	12.2	4.3	11.8	4.1	9.4	4.1
Oct	5.1	3.7	7.1	4.7	6.3	4.6	6.7	3.2	7.7	2.6
Nov	−3.0	6.1	2.8	4.6	3.1	2.8	1.6	3.0	2.9	3.1
Dec	−11.9	5.5	0.0	5.3	−0.2	3.9	−2.2	5.5	1.7	2.2

**Table 10:** Morphed mean monthly temperatures $T_{mean}$, and standard deviation of monthly daily mean temperatures $\sigma_{T}$ in RCP4.5 RCM (ECEM) in 2050. Complete hourly dataset available in [15].

RCP4.5 - RCM										
	S1		S2		S3		S4		S5	
	$T_{mean}$	$\sigma_{T}$	$T_{mean}$	$\sigma_{T}$	$T_{mean}$	$\sigma_{T}$	$T_{mean}$	$\sigma_{T}$	$T_{mean}$	$\sigma_{T}$
	[${}^{\circ }$C]	[${}^{\circ }$C]	[${}^{\circ }$C]	[${}^{\circ }$C]	[${}^{\circ }$C]	[${}^{\circ }$C]	[${}^{\circ }$C]	[${}^{\circ }$C]	[${}^{\circ }$C]	[${}^{\circ }$C]
Jan	−14.1	6.0	−5.7	5.9	−5.7	5.4	−5.5	5.9	−1.5	3.9
Feb	−9.6	6.0	−4.3	5.0	−4.8	5.3	−4.0	5.1	−0.4	3.3
Mar	−2.5	5.5	−0.7	4.2	−1.6	4.3	−0.6	4.0	−1.2	4.1
Apr	5.0	3.3	4.4	4.2	4.5	4.9	4.9	4.4	5.4	4.8
May	12.2	6.4	11.3	4.8	10.8	5.3	10.8	5.7	9.9	5.6
Jun	14.2	4.3	14.9	4.1	14.6	4.0	14.9	5.1	13.8	4.4
Jul	22.0	4.7	17.8	3.8	16.6	4.3	17.1	4.2	16.0	4.1
Aug	16.5	5.6	15.7	4.2	15.2	3.5	15.2	4.7	13.5	3.3
Sep	10.4	3.7	10.3	5.0	11.2	3.8	10.6	3.7	8.4	3.7
Oct	4.2	3.6	5.7	4.6	5.3	4.5	5.6	3.1	6.8	2.6
Nov	−3.4	5.8	1.1	4.6	1.7	2.8	0.3	3.0	1.5	3.1
Dec	−12.3	5.6	−1.9	5.5	−1.5	4.1	−3.7	5.8	0.3	2.3

**Table 11:** Morphed mean monthly temperatures $T_{mean}$, and standard deviation of monthly daily mean temperatures $\sigma_{T}$ in RCP8.5 RCM (ECEM) in 2050. Complete hourly dataset available in [15].

RCP8.5 - RCM										
	S1		S2		S3		S4		S5	
	$T_{mean}$	$\sigma_{T}$	$T_{mean}$	$\sigma_{T}$	$T_{mean}$	$\sigma_{T}$	$T_{mean}$	$\sigma_{T}$	$T_{mean}$	$\sigma_{T}$
	[${}^{\circ }$C]	[${}^{\circ }$C]	[${}^{\circ }$C]	[${}^{\circ }$C]	[${}^{\circ }$C]	[${}^{\circ }$C]	[${}^{\circ }$C]	[${}^{\circ }$C]	[${}^{\circ }$C]	[${}^{\circ }$C]
Jan	−13.6	5.7	−5.2	5.7	−5.2	5.2	−5.0	5.8	−0.8	3.7
Feb	−9.3	5.8	−3.9	4.7	−4.6	5.2	−3.6	4.9	0.0	3.2
Mar	−2.0	5.4	0.0	4.0	−1.1	4.2	0.1	3.8	−0.5	4.1
Apr	5.4	3.2	4.9	4.0	4.9	4.6	5.3	4.3	5.8	4.5
May	12.4	6.1	11.4	4.5	10.9	5.0	10.9	5.3	10.1	5.3
Jun	14.7	4.5	15.3	4.2	15.1	4.2	15.3	5.2	14.4	4.5
Jul	22.5	4.8	18.4	3.9	17.1	4.4	17.6	4.3	16.5	4.2
Aug	16.9	5.4	16.1	4.1	15.6	3.4	15.6	4.5	13.9	3.2
Sep	11.1	3.6	10.9	4.8	11.9	3.7	11.2	3.6	9.1	3.6
Oct	5.1	3.7	6.7	4.5	6.2	4.6	6.6	3.0	7.6	2.6
Nov	−3.0	6.1	1.7	4.8	2.0	2.9	0.9	3.1	1.9	3.3
Dec	−11.9	5.5	−1.2	5.3	−1.1	4.0	−3.1	5.5	0.7	2.2

The morphed outdoor temperature files are stored in Zenodo Repository [15] under Creative Commons Licence 4.0 (CC4.0). These morphed outdoor temperatures were used in [1] to simulate future thermal energy demand in buildings.

## 2 Experimental Design, Materials and Methods

### 2.1 Methodology: morphing

The current weather data is transformed to represent a future climate with the help of a statistical down-scaling method called morphing [14]. The simulated future daily and monthly outdoor temperatures from GCMs and RCMs are statistically down-scaled to hourly level either with shifting, stretching or a combination of shifting and stretching by using hourly data from existing climate. To include both the changes in monthly average temperature and the daily temperature variation, the combination of shifting and stretching method for morphing the outdoor temperature is used:

(1) $$T=T_{0}+\Delta T_{m}+\alpha_{m}\times \left(T_{0}-<T_{0}{>}_{m}\right)$$

where T is the morphed temperature [°C], $T_{0}$ is the hourly baseline temperature [°C], $\Delta T_{m}$ is the change in monthly average temperature [°C], $\alpha_{m}$ is the fractional change in monthly temperature [-] and $<T_{0}{>}_{m}$ is the monthly average temperature in baseline scenario [°C] [14]. An averaging period of 30 years is used in all $\Delta T_{m}$ and $\alpha_{m}$ calculations to reduce the impact of a yearly variations in the used datasets.

The fractional change $\alpha_{m}$ is used to asses the change in daily temperature variation, and it can be calculated in 2 ways depending on the available data. The first method is to utilize the changes in daily maximum and minimum temperatures if this data is available:

(2) $$\alpha_{m}=\frac{\Delta T_{MAX,m}-\Delta T_{MIN,m}}{<T_{MAX,m}>-<T_{MIN,m}>}$$

where $\Delta T_{MAX,m}$ is the change in average daily maximum temperature in month m [°C], $\Delta T_{MIN,m}$ is the change in average daily minimum temperature in month m [°C], $<T_{MAX,m}>$ is the average daily maximum temperature in month m [°C] and $<T_{MIN,m}>$ is the average daily minimum temperature in month m [°C] [14].

The second method to calculate $\alpha_{m}$ follows method M2 from [16]. Here it is assumed that the daily temperature variation is relative to the daily mean temperature variation on a certain month in case no data on daily maximum and minimum temperatures is available:

(3) $$\alpha_{m}=\frac{\sigma_{T,new}}{\sigma_{T,0}}-1$$

where $\sigma_{T,new}$ is the new daily variance on month m [-] and $\sigma_{T,0}$ is the daily mean temperature variance on the baseline scenario on month m [-] [17].

**Fig. 4:**
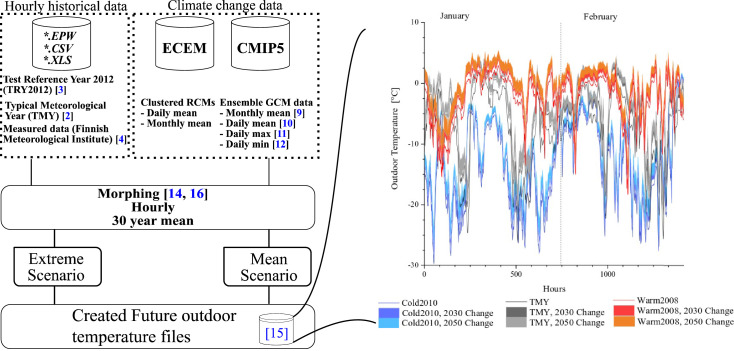
The procedure for creating future outdoor temperature data, showing the input data sources, the methodology, scenarios and the created data (modified from [1]).

The whole procedure of creating the hourly future outdoor temperature data is presented in Fig. 4. It shows the 2 sources of input data, from which the hourly data is used as the baseline data, and the climate change data is used to calculate the change in the monthly average temperature $\Delta T_{m}$ from monthly data and the fractional change $\alpha_{m}$ from the daily data depending on the type of climate change data that is available. The morphing procedure is then used according to Equation 1 - Equation 3. This results in the morphed future outdoor temperatures on hourly-scale, which can represent either future extreme or mean weather scenario depending on the used baseline data.

The morphing was conducted with a weather morphing application that was created in MATLAB environment to calculate the described variables from existing weather data files and from climate change projection results from the GCM and RCM simulations. These variables are then used to create new morphed outdoor temperatures based on the desired baseline scenario, morphed year and morphing period. The created weather morphing application allows utilizing both daily and monthly change data and supports both calculation methods for the calculation of fractional change $\alpha_{m}$. The application is an open-source model and available at GitHub repository.^2^

## CRediT Author Statement

**Jari Pulkkinen:** Methodology, Software, Formal analysis, Investigation, Data curation, Writing – original draft, Writing – review & editing, Visualization; **Jean-Nicolas Louis:** Conceptualization, Software, Validation, Formal analysis, Data curation, Writing – original draft, Writing – review & editing, Visualization, Supervision, Project administration, Funding acquisition.

## Declaration of Competing Interest

The authors declare that they have no known competing financial interests or personal relationships which have, or could be perceived to have, influenced the work reported in this article.

## Appendix Supplementary materials

Image, application 1

## Footnotes

2 https://github.com/japulk/Weather-Morphing-Tool

## Footnotes

1 http://ecem.wemcouncil.org
