# Supplementary figures and images for: Near- and medium-term hourly morphed mean and extreme future temperature datasets for Jyväskylä, Finland, for building thermal energy demand simulations
Source: Data Brief. 2021 Jun 10;37:107209. doi: 10.1016/j.dib.2021.107209 (PMC8215183; doi:10.1016/j.dib.2021.107209)

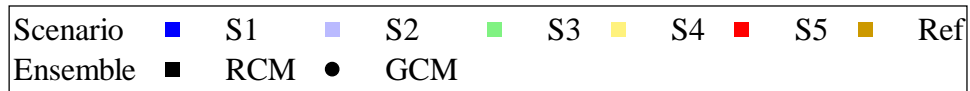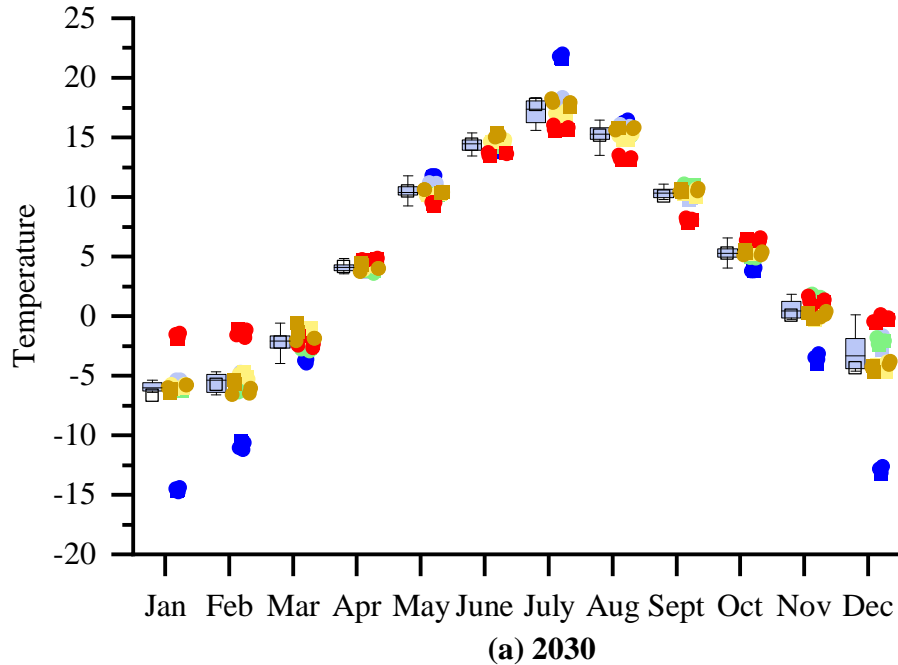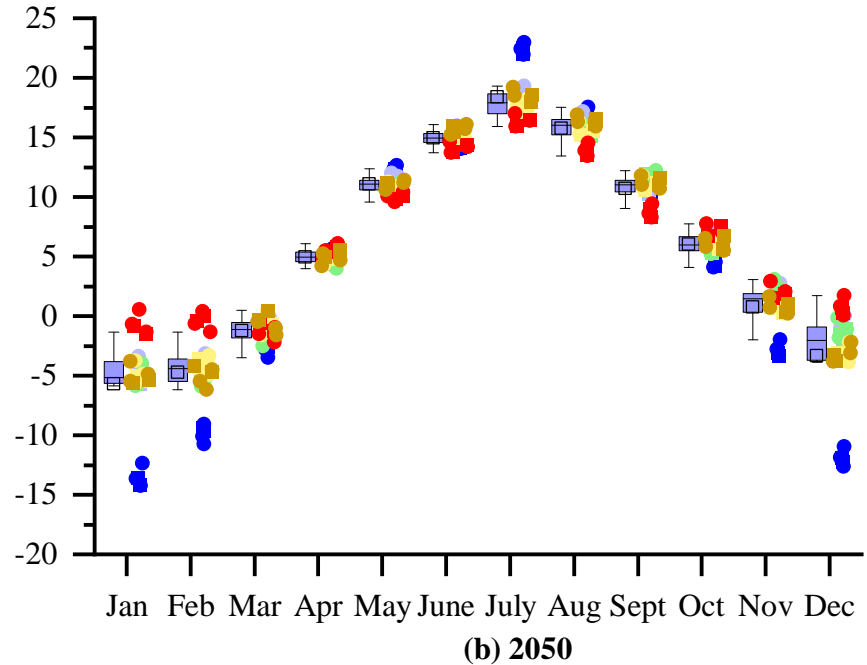

Supplement: Supplementary file 1 [file mmc1.pdf]
